# Versatility of Approximating Single-Particle Electron Microscopy Density Maps Using Pseudoatoms and Approximation-Accuracy Control

**DOI:** 10.1155/2016/7060348

**Published:** 2016-12-21

**Authors:** Slavica Jonić, Carlos Oscar S. Sorzano

**Affiliations:** ^1^IMPMC, Sorbonne Universités, CNRS UMR 7590, UPMC Univ Paris 6, MNHN, IRD UMR 206, 75005 Paris, France; ^2^Biocomputing Unit, Centro Nacional de Biotecnología, CSIC, Campus de Cantoblanco, Darwin 3, 28049 Madrid, Spain

## Abstract

Three-dimensional Gaussian functions have been shown useful in representing electron microscopy (EM) density maps for studying macromolecular structure and dynamics. Methods that require setting a desired number of Gaussian functions or a maximum number of iterations may result in suboptimal representations of the structure. An alternative is to set a desired error of approximation of the given EM map and then optimize the number of Gaussian functions to achieve this approximation error. In this article, we review different applications of such an approach that uses spherical Gaussian functions of fixed standard deviation, referred to as pseudoatoms. Some of these applications use EM-map normal mode analysis (NMA) with elastic network model (ENM) (applications such as predicting conformational changes of macromolecular complexes or exploring actual conformational changes by normal-mode-based analysis of experimental data) while some other do not use NMA (denoising of EM density maps). In applications based on NMA and ENM, the advantage of using pseudoatoms in EM-map coarse-grain models is that the ENM springs are easily assigned among neighboring grains thanks to their spherical shape and uniformed size. EM-map denoising based on the map coarse-graining was so far only shown using pseudoatoms as grains.

## 1. Introduction

Single-particle analysis is an electron microscopy (EM) technique that allows determining the structure at near-atomic resolutions for a large range of macromolecular complexes [1–16]. Also, it allows studying conformational variability of macromolecular complexes by determining their different conformations [17–22]. These different conformations are usually obtained by analyzing heterogeneity with methods that assume a small number of discrete conformations coexisting in the specimen [23–28], while several methods have been recently developed to help analyzing continuous conformational changes [29–33].

EM-map representations with a reduced number of points or with a set of 3D Gaussian functions have been shown useful in studying macromolecular structure and dynamics [30, 33–44]. The process of representing EM maps with a set of points or 3D Gaussian functions (grains) is sometimes referred to as coarse-graining of EM maps. A typical approach to coarse-graining is a neural network clustering approach that quantizes the given EM map so that the probability density of the grains closely resembles the probability density of the given data, which makes the coarse-grain representation retain the overall shape of the structure from the given EM map [34, 36, 38–40]. This approach is referred to as Vector Quantization (VQ). A different approach is to parametrize a Gaussian Mixture Model (GMM) of the probability density function using expectation-maximization algorithm [41, 45]. All these approaches require setting a desired (target) number of grains or a maximum number of iterations to stop the iterative procedure, which may result in suboptimal representations. Indeed, the use of a small target number of grains or a small maximum number of iterations may lead to a small final number of grains resulting in a model with overrepresented high density regions and underrepresented low density regions. Furthermore, in the case of symmetrical structures, the inadequately small final number of grains can result in representations that are overall nonuniform (asymmetrical). A difficulty is thus to choose the stopping parameter that will produce a sufficiently high number of grains to appropriately represent all density regions.

An alternative is to set a desired (target) error of approximation of the given EM map and then optimize the number of Gaussian functions, their position, and their weights to achieve the target approximation error, as in the approach that we introduced in [43]. In each iteration, this approach adds some Gaussian functions (grains) while removing some (the grains with small weights or distances will be removed). We have found that this strategy of minimizing the global representation error, involving controlled adding and removing grains, allows placing new grains where they are most needed and adapting the grains near the removed ones to better represent the local intensity in the input EM map [43], which helps overcoming the underrepresentation problem. For instance, we have found that symmetry is preserved in EM-map approximations with this strategy for typical values of the target approximation error such as 1–15% [30, 33, 42–44]. This method uses spherical Gaussian functions of fixed standard deviation that we refer to as pseudoatoms. Its versatility has been shown in applications such as predicting conformational changes of macromolecular complexes, exploring actual conformational changes, analyzing continuous conformational changes, and denoising of EM density maps [30, 33, 42–44]. Some of these applications are based on EM-map normal mode analysis (NMA) with elastic network model (ENM) [46, 47] (e.g., predicting conformational changes of macromolecular complexes or exploring actual conformational changes using normal-mode-based analysis of experimental data). In some other applications, NMA is not used (e.g., denoising of EM density maps).

The advantage of using pseudoatoms in applications based on NMA and ENM, with respect to other types of grains (Table 1), is their uniformity over the molecule that is a prerequisite for a simple application of the ENM. Indeed, as pseudoatoms have spherical shape and uniformed size over the molecule, they allow an easy setting of springs among neighboring grains in the ENM. On the contrary, 3D points (the so-called codebook vectors) obtained with VQ can be regarded as Gaussian functions whose standard deviation can vary over the molecule (each codebook vector is associated with a data subregion known as Voronoi cell [34] and different subregions can have different sizes), which may make the ENM setting more complicated than with Gaussian functions of the same standard deviation. Similarly, NMA should be more complicated with ellipsoidal Gaussian distribution obtained with the GMM approach (to the best of our knowledge, such NMA has not been reported so far). Regarding EM-map denoising (not based on NMA), only pseudoatoms were so far reported as grains in that application of coarse-graining [44].

In this article, we review the mentioned applications of this EM-map approximation method while only briefly reminding the method. For algorithmic details (e.g., related to adding/removing grains), the reader is addressed to [43] that describes this method in detail.

## 2. Background

We start this section with a brief background on the approach for converting EM maps in sets of pseudoatoms. Then, we provide a brief reminder on NMA that is used in applications of the conversion approach to studying conformational changes of macromolecular complexes.

### 2.1. Approximation of EM Maps Using Pseudoatoms and Approximation Error Control

A function *f*(**r**)  (**r** ∈ *ℝ* ^3^) can be approximated using Gaussian radial basis functions (RBFs) by ${\hat{f}}_{N}\left(r\right)=\sum_{i=1}^{N}\omega_{i}K_{\sigma }\left(\left\|r-r_{i}\right\|\right)$, where *K*_*σ*_(*r*) is the RBF kernel that is a Gaussian function with the standard deviation *σ* and the amplitude of 1, to which we refer as pseudoatom, *N* is the number of pseudoatoms, **r**_*i*_ is the vector of the center coordinates of the *i*th pseudoatom, ‖**r** − **r**_*i*_‖ is the Euclidean distance between the vectors **r** and **r**_*i*_, and *ω*_*i*_ > 0 is the weight (contribution) of the *i*th pseudoatom. Given an EM density map *f*(**r**)  (**r** ∈ *ℝ* ^3^), a Gaussian-function standard deviation *σ*, and a target approximation error *ε*, our approach determines the number of pseudoatoms *N*, their positions **r**_*i*_, and weights *ω*_*i*_ such that the approximation error *e*_*N*_ satisfies $e_{N}=\left(1/V\right)\sum_{j=1}^{V}\left(\left|f\left(r_{j}\right)-{\hat{f}}_{N}\left(r_{j}\right)\right|/\Delta f\right)<\varepsilon$. Here, Δ*f* is the effective range of values in the EM map, **r**_*j*_ is the voxel location at which the given EM map is compared with its approximation, and *V* is the total number of evaluated voxels (the evaluation can be done in a region of interest defined by a mask). To avoid getting trapped into local minima of the error *e*_*N*_, new pseudoatoms are added progressively in regions with large errors and weights and positions of the current number of pseudoatoms are determined by a gradient descent minimization of *e*_*N*_. It should be noted that pseudoatom positions **r**_*i*_ do not necessarily coincide with voxel positions **r**_*j*_ because the pseudoatom positions vary continuously within the EM density map. Also, it should be noted that *σ* can be expressed in angstroms, but it is here expressed in voxels. The typical values of *σ* are between 1 and 2 (voxels).

**Figure 1:**
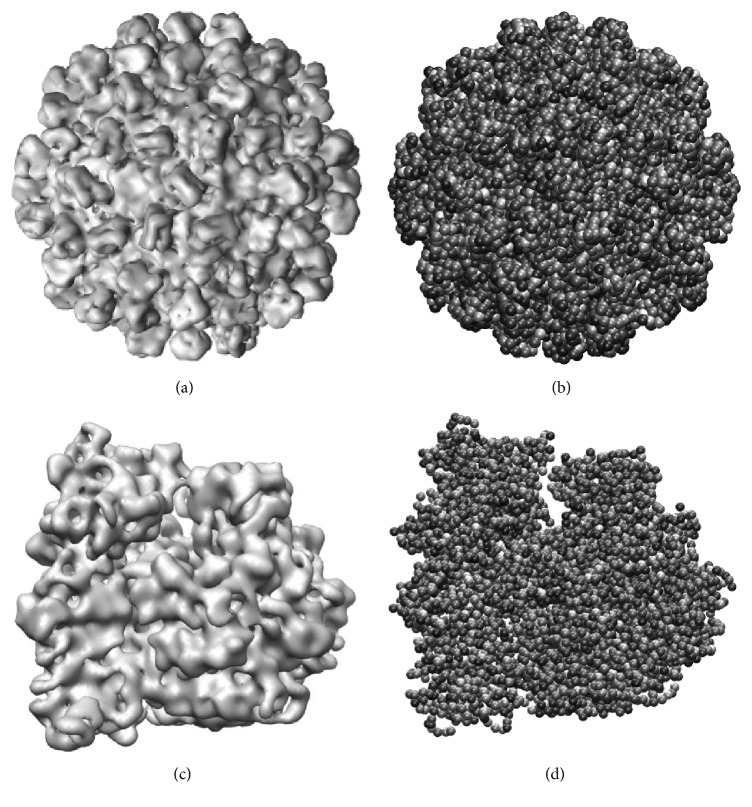
Approximation of density maps to a desired level of accuracy (*ε*) using 3D Gaussian functions of a given standard deviation (*σ*). (a, b) Synthetic density map of Tomato Bushy Stunt Virus at 15 Å resolution from an atomic structure available in PDB (the structure from [55]) (a) and its pseudoatomic representation using *σ* = 1.3 and *ε* = 4% (b). (c, d) Synthetic density map of 70S ribosome at 15 Å resolution from an atomic structure available in PDB (the structure from [56]) (c) and its pseudoatomic representation using *σ* = 1 and *ε* = 2% (d). In this figure, 3D Gaussian functions (referred to as pseudoatoms) are shown as spheres of a radius related to *σ* and overlapping spheres are used for a nicer visualization of the pseudoatomic structure.

Different pseudoatom representations can be obtained with this approach by varying the Gaussian-function standard deviation (*σ*) and the target approximation error (*ε*) (Figure 1). Smaller values of *σ* and *ε* result in larger numbers of pseudoatoms and vice versa. However, *σ* and *ε* should be chosen taking into account resolution and noise of the given EM map (e.g., large *σ* and *ε* may give sufficiently good approximations of low resolution maps) and the target application, as we show here.

### 2.2. Normal Mode Analysis

NMA models complex motions by linear combinations of harmonic oscillations around a minimum-energy conformation. NMA is often based on the standard ENM [46], which is also the case in our approach. More precisely, we perform NMA of pseudoatom representations of EM maps using the software developed by Tama et al. [47]. In this approach, the given EM map is assumed to contain the structure in the minimum-energy conformation and no energy minimization is required [46]. It has been shown that NMA of EM density maps of intermediate resolution result in a good approximation of normal modes of atomic-resolution structures, in particular at low frequencies at which the motions were experimentally observed [47]. An experimentally observed motion here means a transition between two different conformations of the same complex obtained at atomic resolution (e.g., by X-ray crystallography). This transition and the conformational states along it can be modeled (simulated) using low-frequency normal modes of any of the two given conformations. For instance, the low-frequency modes having the highest overlap with the difference between the two given atomic-resolution conformations usually capture 60–70% of the conformational change [47, 48]. Normal modes of EM density maps can be used to obtain other possible conformational states of the same complex (to predict low-frequency motions of the complex). Note here that the conformational states are discrete and can be regarded as discrete samples of a continuous trajectory of conformational transition. NMA of intermediate-resolution EM density maps is especially useful when atomic-resolution structures cannot be obtained [37, 38, 47], but it requires coarse-grain representations of the density maps.

Nodes of the elastic network model are 3D point particles. Each node is connected, via harmonic springs, with other nodes within a sphere of a given radius (the radius is referred to as interaction cutoff distance). In our approach, the coordinates of nodes of the ENM are the center coordinates of pseudoatoms with which the given EM density map is represented. Given *N* nodes of the ENM, NMA requires a diagonalization of a 3*N* × 3*N* matrix of second derivatives of the potential energy (Hessian matrix), which is performed via eigenanalysis of the Hessian matrix. Normal modes are eigenvectors of the Hessian matrix while the normal mode frequencies are the square roots of eigenvalues of the Hessian matrix.

**Figure 2:**
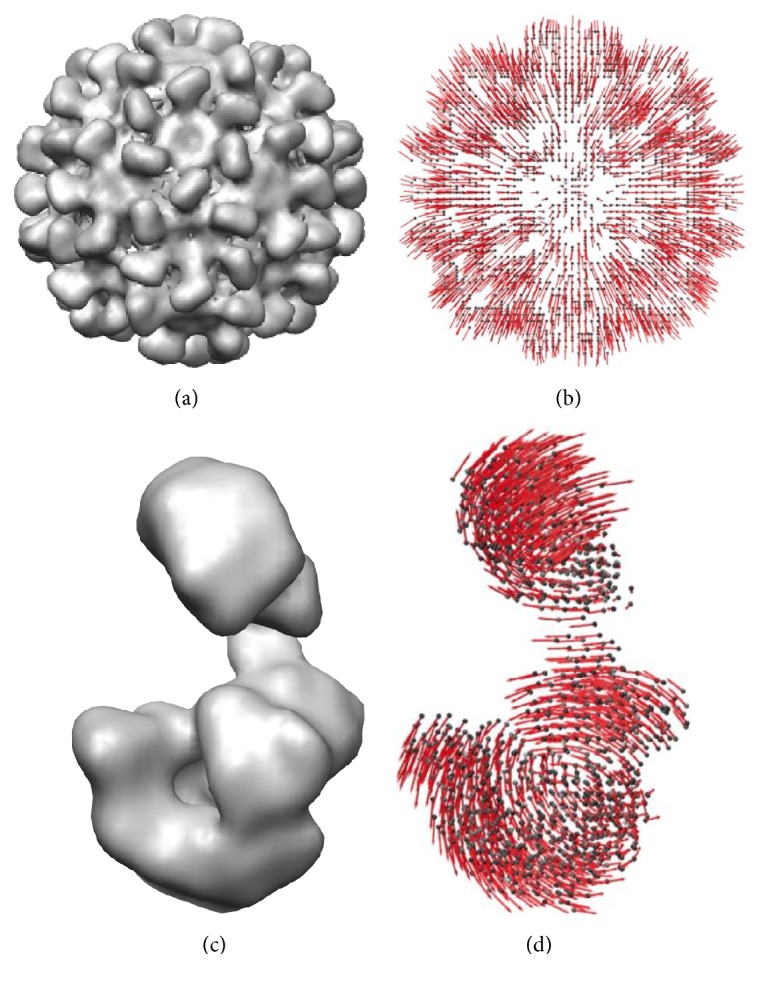
Normal modes of pseudoatomic structures from EM density maps for simulations of structural flexibility. (a, b) EM density map of a compact conformation of Tomato Bushy Stunt Virus from [57] (a) and a displacement of pseudoatoms along the normal mode describing the swelling motion of the virus capsid that has also been observed experimentally [57, 58] (b). (c, d) EM density map of a DNA polymerase Pol *α*-B subunit complex of the eukaryotic primosome from [59] (c) and a displacement of pseudoatoms along the normal mode describing the bending-unbending motion that has also been observed experimentally [30, 59] (d). In this figure, very small spheres are used to represent 3D Gaussian functions (referred to as pseudoatoms) and not all spheres are shown in order to avoid their overlapping and allow a nicer visualization of the displacement of pseudoatoms along the particular normal mode.

A displacement of nodes of the ENM along normal modes modifies the given conformation, which is used in simulations of structural flexibility (Figure 2). The coordinates of normal modes are expressed in angstroms while the displacement amplitudes along normal modes have no units. Six lowest-frequency modes are related to rigid-body movements and are usually not used for the displacement.

## 3. Applications

### 3.1. Prediction of Conformational Changes

As explained in Background, NMA of intermediate-resolution EM maps provides normal modes that can be used to simulate (predict) different conformational states of the same complex. Normal modes of EM maps are calculated based on the EM-map coarse-grained representations with pseudoatoms. Both EM-map coarse-graining and NMA can be performed using* 3DEM Loupe* web server [42].

*3DEM Loupe* [42] is currently the only web server that allows interactive NMA of EM density maps. Other web servers perform NMA of atomic-resolution structures (e.g.,* ElNemo* [49]) or do not allow the user to input his/her own EM density map for NMA (e.g.,* EMotion* [38]). The workflow of* 3DEM Loupe* consists of the following three steps: (1) conversion of the input EM density map into a pseudoatomic structure (coarse-graining) based on the approximation error control; (2) calculation of normal modes of the pseudoatomic structure; and (3) animation of the obtained normal modes (animated displacement of pseudoatomic structures along normal modes). The pseudoatomic structure, normal modes, and animations obtained by* 3DEM Loupe* can be downloaded for their further analysis on a local computer. The server also contains precomputed results for several EM maps, such as 70S ribosome, GroEL, ribosome-bound termination factor RF2, and connector of bacteriophage T7.

### 3.2. Exploring Actual Conformational Changes Including Continuous Conformational Changes

Coarse-grain representations of EM density maps and normal modes of those coarse-grain representations can be used to analyze experimental EM data. More precisely, conformations actually present in EM data can be interpreted using simulated conformations (NMA-based simulations). In the framework of conformational heterogeneity analysis, normal modes were used for image analysis first in [50], but the first procedure capable of automatically processing large series of images using as many “test” conformations as needed is* HEMNMA* [30, 51]. In this subsection,* HEMNMA*, the approach for NMA-based conformational analysis of a series of images (using normal modes of a given density map), is presented together with an approach for NMA-based conformational analysis of a series of density maps (*StructMap*).

#### 3.2.1. Analysis of EM Images:* HEMNMA*

**Figure 3:**
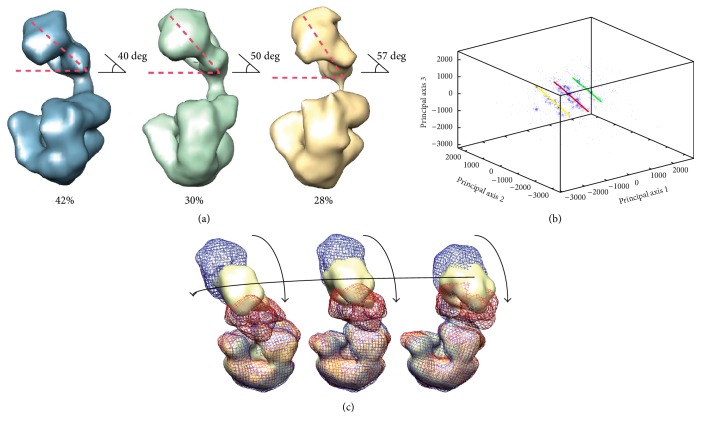
Exploring actual conformational changes by image analysis using normal modes of Gaussian-based (pseudoatomic) representation of EM density maps (analysis with HEMNMA). (a) EM density maps of three conformations of a DNA polymerase Pol *α*-B subunit complex of the eukaryotic primosome showing bending-unbending motion of the complex and the percentage of images assigned to each density map using ML3D, a method that assumes a small number of different coexisting conformations in the specimen, from [59]. (b) Mapping of images used in the analysis in [59] (producing the density maps shown in (a)) onto a low-dimensional distance space based on a flexible 2D-to-3D fitting between the images and a reference density map (the one with the highest percentage of assigned images in [59]) using normal modes of the pseudoatomic representation of the reference density map (in this space, images are represented with points and the distances between the points correspond to the differences between the corresponding conformations). (c) Displacement of the reference pseudoatomic structure along the trajectories identified in the densest regions of the distance space shown in (b), which indicates a bending-unbending motion, detected also by ML3D, and changes in the length of the flexible linker between the two lobes that could not be detected with ML3D (from left to right: the displacement along the yellow, red, and green trajectory shown in (b); the displacement is shown by providing three frames of an animation represented by red, yellow, and blue isosurfaces of the density maps into which the reference pseudoatomic structure was converted during the displacement; the arrows indicate the motions visualized by the frames). Reproduced with permission from [59] (a) and from [30] (b and c).

Given an EM density map,* HEMNMA* [30, 51] computes its pseudoatom representation and performs NMA of the obtained pseudoatomic structure, as does* 3DEM Loupe* [42]. This pseudoatomic structure and its normal modes are then used to analyze each single-particle image by elastic projection matching. More precisely,* HEMNMA* deforms the pseudoatomic structure using some amplitudes of the displacement along normal modes and then compares the image with projections of the pseudoatomic structure converted into a density map. The image is assigned the orientation, translation, and normal mode amplitudes of the best-matching projection. The normal mode amplitudes obtained for all images are finally mapped onto a low-dimensional distance space (usually, 1D, 2D, or 3D), which allows seeing the distribution of conformations. This space can be explored to detect whether the conformational change is discrete (points are grouped in a few clusters) or continuous (absence of clusters, points spread over the space). In the case of continuous conformational changes, conformational trajectories may be identified by analyzing the most populated regions in this space. Along the trajectories, the given EM map and the obtained pseudoatomic structure can be animated and 3D reconstructions can be calculated. It has been shown that this methodology can help detect the motions undetectable with methods that assume a small number of different coexisting conformations in the specimen [30] (Figure 3). In this context, the conformational heterogeneity of* E. coli* 70S ribosome, DNA polymerase Pol *α*-B subunit complex of the eukaryotic primosome, and Tomato Bushy Stunt Virus was described more extensively with* HEMNMA* than with other methods [30].

**Figure 4:**
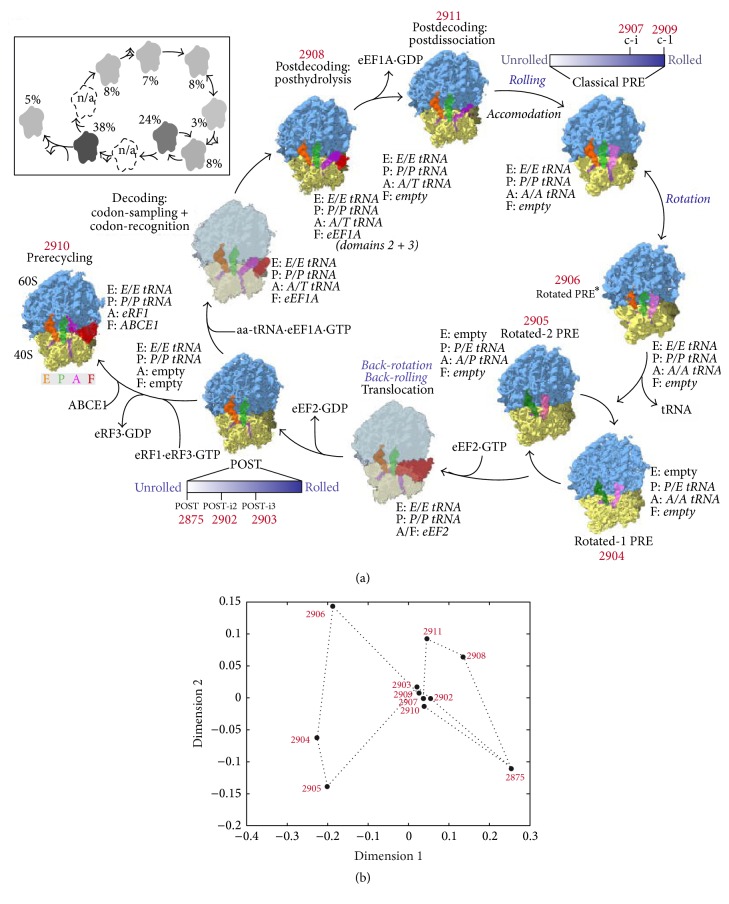
Exploring actual conformational changes by analyzing a set of EM density maps using normal modes of Gaussian-based (pseudoatomic) representation of these maps (analysis with StructMap). (a) States along the elongation cycle of 80S ribosome, from which eleven states were determined by EM in [53] (the EMDB code of each determined density map is provided next to it in red color; translocation and decoding-sampling/recognition states were not experimentally observed in the original work and are shown grayed out and without associated EMDB code or marked with n/a in the inset). (b) Mapping of EM density maps, denoted in (a) with their EMDB codes, onto a low-dimensional distance space based on a flexible 3D-to-3D fitting between the density maps using normal modes of their pseudoatomic representations (in this space, the density maps are represented with filled circles and EMDB codes and the distances between the points correspond to the conformational differences that remain after the flexible fitting). Dotted lines are used in (b) to connect subsequent states along the cycle shown in (a). Slightly modified reproductions from [53] (a) and [33] (b), with permission.

It should be noted that* HEMNMA* can also analyze a series of images using an atomic-resolution structure instead of a density map. It should also be noted that the conformational variability shown in Figure 3(b) is rarely seen in EM because the majority of computational methods assume specimens with few different conformations of complexes (e.g., Figures 3(a) and 4(a)). Additionally, biochemical specimen preparation protocols are usually optimized so as to reduce the number of different conformations in the specimen. To achieve atomic resolution of reconstruction, the computational methods are usually based on different rounds of 2D and 3D classifications (involving computations of average particle views and conformations), during which many particle images are removed and only particles assigned to high-resolution classes (particles with most consistent views and conformations) are kept for final 3D reconstructions. Such biochemical and computational “selection” of particles may obscure full conformational variability of the complex. On the contrary, HEMNMA method estimates the conformational variability distribution (e.g., Figure 3(b)) using raw images and no classification, which facilitates deciphering the full range of conformational variability that is a quasicontinuum of conformational states (a large number of discrete samples of a continuous conformational transition trajectory). Analysis of continuous conformational transitions by EM is currently a research field in expansion [29–32, 52], which will be reviewed in a separate publication.

#### 3.2.2. Analysis of EM Maps:* StructMap*

*StructMap* [33] automatically analyzes a set of EM density maps to map them onto a common low-dimensional distance space (usually, 1D, 2D, or 3D). It requires representing each density map with pseudoatoms and computing normal modes of each obtained pseudoatomic structure. The actual conformation in each EM density map (reference map) is estimated by elastic fitting of this map with the pseudoatomic structures of all other maps from the given set of EM maps. More precisely, pseudoatoms are displaced along normal modes and the displacement amplitudes are adjusted to maximize the cross-correlation coefficient between the map calculated from displaced pseudoatoms and the reference map. The obtained maximum cross-correlation coefficients are subtracted from 1 and then mapped onto a low-dimensional space, which shows the differences (distances) among given EM density maps that cannot be interpreted using normal modes. In the case of continuous conformational distribution, EM density maps can be regarded as discrete, unordered samples of continuous trajectories and* StructMap* can be used to get a rough idea of these trajectories (potential sequences of conformational changes) [33]. In some heterogeneity cases, such as a combined conformational and compositional heterogeneity of states along the elongation cycle of human 80S ribosome [53], this task can be particularity challenging, but* StructMap* can still help visualizing the differences between the states and analyzing potential trajectories [33] (Figure 4).

In the context of revealing sequences of conformational changes,* StructMap* can also be combined with* HEMNMA*. For instance, instead of using* HEMNMA* to analyze all images with all given EM density maps, a few EM maps can be chosen from the distance space obtained by* StructMap* so as to explore more finely particular regions in this space.

### 3.3. Denoising

Cryo-EM images have a low signal-to-noise ratio. Thus, a large number of such images must be averaged in 3D space to reduce noise. However, even high-resolution 3D reconstructed EM maps may still contain significant amounts of noise. We have shown that pseudoatom representations of EM density maps can be used for EM-map denoising [44]. We assume that the object reconstructed from images is correct enough, meaning that effects of noise dominate potential reconstruction artifacts and potential effects of heterogeneity of images that were used for this reconstruction. Smaller target approximation errors (*ε*) and smaller Gaussian standard deviations (*σ*) generally produce better approximations of EM maps (i.e., the density maps computed from pseudoatomic structures have more details), with a risk of reproducing the EM-map noise in the density map computed from pseudoatoms. Setting *ε* or *σ* to higher values can reduce this risk. These two parameters should be set in accordance with the resolution of the given EM map. We have shown that *σ* = 1.5 (voxels) and *ε* = 1% produce satisfactory denoising results in most cases of higher-resolution density maps (resolutions higher than 6 Å, gold-standard FSC 0.143), whereas denoising of lower-resolution density maps is usually achieved using larger values of *ε* (*ε* = 5%–15%) and the values of *σ* that may need to be adjusted around its default value (*σ* = 1.5) [44].

**Figure 5:**
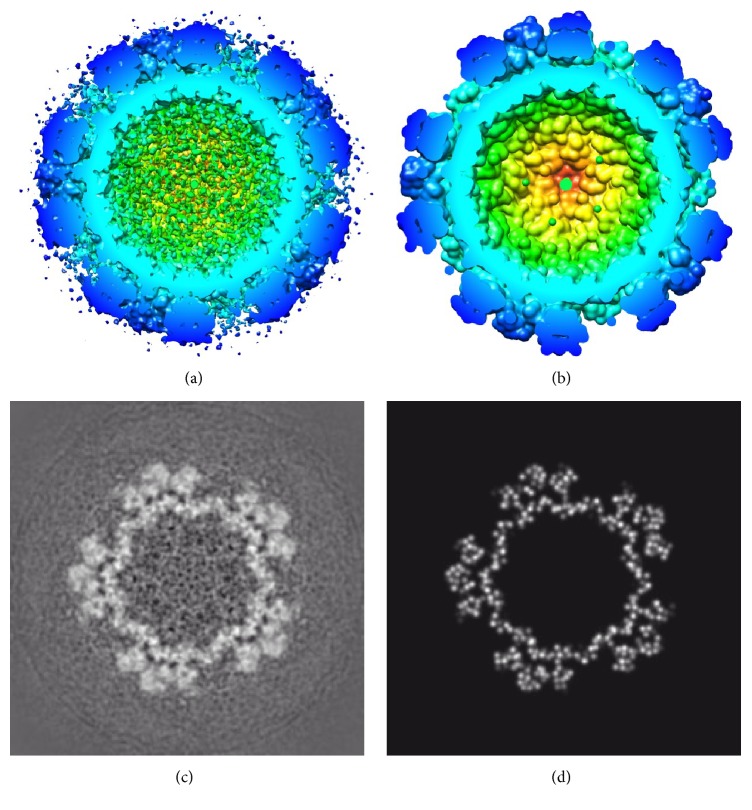
Denoising of EM density maps based on their approximation to a desired level of accuracy (*ε*) using 3D Gaussian functions of a given standard deviation (*σ*). (a and b) Half of the density map of genogroup II genotype 10 norovirus virus-like particle from [60] radially colored with Chimera before (a) and after (b) denoising (denoising based on *σ* = 1.5 and *ε* = 15%). (c and d) Density-map arbitrary slice before (c) and after (d) denoising, for the density maps shown in (a) and (b), respectively. Reproduced with permission from [44].

The method was used to denoise EM density maps of several complexes obtained at subnanometer resolutions by single-particle analysis or subtomogram averaging (beta-galactosidase, ribosome, and empty and full virus particles) [44] (Figure 5).

## 4. Discussion

The method for EM-map approximation using Gaussian functions of standard deviation *σ* and amplitude 1 and using a target approximation error *ε* [43] has applications in predicting conformational changes of macromolecular complexes, exploring actual (discrete or continuous) conformational changes, and denoising of EM density maps. In this article, we reviewed these applications, together with the software that uses this method and the examples of results that can be obtained with this software.

In this approach, the EM-map approximation is a weighted sum of Gaussian functions of given standard deviation *σ* and amplitude 1 (referred to as pseudoatoms) whose number, location, and weights are determined by minimizing the EM-map approximation error towards the given target error *ε*. The shape and density distribution of a given macromolecular complex are thus fully represented with the distribution of Gaussian functions. The centers of pseudoatoms are control points that can be displaced (e.g., along normal modes) to modify a given conformation of the complex. The modified conformation, represented with the pseudoatoms, can be converted into a density map by computing the weighted sum of Gaussian functions at new locations (the same weight as before the modification).

The target approximation error control allows different applications of this method. We could learn from experiments how to choose this target approximation error to suit these different applications. For instance, in EM-map denoising applications, target approximation errors smaller than 5% are recommended for EM maps of higher resolution (higher than 6 Å according to the gold-standard FSC 0.143 criterion), whereas larger target approximation errors (5%–15%) are recommended for EM maps of lower resolution [44]. In applications such as simulations of conformational changes (NMA of EM density maps) or elastic fitting among several EM maps (elastic 3D-to-3D fitting), it is recommended to use target errors of 10%–15% with EM maps that are noisy or that have different (low and high) resolutions among each other, whereas target errors of 1%–5% are recommended with clean and high-resolution EM maps [33, 42]. In elastic 3D-to-2D fitting applications, smaller target errors (1%–5%) are recommended to guarantee a good quality of projections of the density map from pseudoatoms (a good projection quality is required for an accurate projection matching with images) [30]. In these different applications, the standard deviation of Gaussian functions is usually in the range between 1 and 2 (voxels) and the value of 1.5 (voxels) can often be chosen as a default value, though adjustments are required around this value to achieve optimal results (quality can be evaluated by comparing target and achieved approximation errors).

Advantages of structural representations with pseudoatoms have recently been explored in the context of 3D reconstruction. For instance, a Bayesian approach to ab initio, low resolution 3D reconstruction proposed in [54] is based on estimating a pseudoatomic model using a random model for initialization and a given number of pseudoatoms. It should be noticed that the Gaussian-function standard deviation in that approach is estimated together with other parameters of the pseudoatomic model (position and weights of pseudoatoms) as well as the parameters of image orientation and translation [54]. That approach makes calculations using class averages instead of raw images and it does not consider a possible conformational heterogeneity of the given set of images. We are currently working on a method based on pseudoatoms with a fixed Gaussian-function standard deviation to iteratively refine a preliminary model taking into account conformational heterogeneity of the data set. This new method should result in a 3D reconstruction (model) optimally representing all given (raw) images via the determined elastic (normal-mode-based) transformations between the model and the images.

## Figures and Tables

**Table 1:** Comparison of Vector Quantization, Gaussian Mixture Model, and our pseudoatomic model.

	Vector Quantization	Gaussian Mixture Model	Our pseudoatomic model
Grain	3D point (codebook vector)	3D Gaussian distribution function	3D radial basis function (isotropic Gaussian distribution function)
Grain geometry	Spherical	Ellipsoidal	Spherical
Algorithm	Self-organizing map (SOM)	Maximum likelihood method using the expectation maximization algorithm	Iterative adding and removing of pseudoatoms and gradient descent refinement
Goal of algorithm	Minimize the mean-square deviation of the codebook vectors from the corresponding 3D data	Find the model with the maximum likelihood function	Find the model with the minimum number of grains for the given error of density approximation
Number of grains	Fixed	Fixed	Adjustable
Grain weight	Adjustable	Adjustable	Adjustable
Grain position	Adjustable	Adjustable	Adjustable
Grain size	Adjustable	Adjustable	Fixed
Application of elastic network model	Easy	Difficult	Easy
